# Caudatin blocks the proliferation, stemness and glycolysis of non-small cell lung cancer cells through the Raf/MEK/ERK pathway

**DOI:** 10.1080/13880209.2022.2050768

**Published:** 2022-04-06

**Authors:** Juan Hou, Qing Chen, Yufeng Huang, Zhiwei Wu, De Ma

**Affiliations:** Department of Oncology, Jingjiang People’s Hospital, Taizhou, Jiangsu, China

**Keywords:** Traditional Chinese medicine, NSCLC, signal pathway, *Cynanchum auriculatum*

## Abstract

**Context:**

The antitumor effects of caudatin have been explored in multiple cancers, but the research on lung cancer has not been fully understood.

**Objective:**

We explored the effects of caudatin on non-small cell lung cancer (NSCLC) *in vitro* and *in vivo*.

**Materials and methods:**

In the *in vitro* experiments, 0, 25, 50 and 100 μM of caudatin were selected to examine the effects on stemness and glycolysis. Subcutaneous tumour xenografts were constructed by injecting the nude mice (BALB/C) with 5 × 10^6^ H1299 cells. In the *in vivo* experiments, all nude mice were divided into the caudatin group (50 mg/kg/day, *n* = 5) and the sham group (equal amount of DMSO, *n* = 5).

**Results:**

The IC_50_ of caudatin for H1299 and H520 cells was 44.68 μM and 69.37 μM, respectively. Compared with caudatin 0 μM group, cell apoptosis rate was increased about 10 times and cell stemness was decreased by 75–85% in caudatin 100 μM group. Glucose uptake (65–80% reduction), lactic acid production (75–80% reduction), ATP level (70–80% reduction) and the expression of HK2 and LDHA (75–85% reduction) were decreased in caudatin 100 μM group. The expression of Raf/MEK/ERK pathway related proteins was decreased to 20–25% by caudatin. Tumour weight (about 70% reduction) and the expression of stemness, glycolysis and Raf/MEK/ERK pathway related proteins (about 50–75% reduction) were suppressed by caudatin *in vivo*.

**Discussion and conclusions:**

We revealed that caudatin blocked stemness and glycolysis in NSCLC for the first time. More experiments about exact dosage of caudatin *in vivo* should be conducted.

## Introduction

A survey of 36 malignant tumours in 185 countries and regions found out that the mortality of lung cancer accounted for 1/5 of all malignant tumour deaths (Ferlay et al. 2019). Non-small cell lung cancer (NSCLC) accounts for about 85% of all the lung cancer cases (He et al. 2020). At present, the curative effects of surgery, radiotherapy and chemotherapy are insufficient due to individual differences, drug resistance and radiotherapy resistance. In addition to external interference therapy, interference on tumour cell metabolism acts as a promising direction of tumour therapy.

Conventional radiotherapy and chemotherapy not only destroy tumour cells, but also bring serious side effects to normal cells. Traditional Chinese medicine (TCM) used in cancer treatment has the advantages of extensive resources, low cost, low toxicity and fewer side effects (Liao et al. 2017). With the development of drug screening and gene therapy technology, the combination of TCM with gene therapy has gradually become a new therapy direction (Qi et al. 2015). Caudatin is a C-21 steroidal aglycone extracted from ‘baishouwu’, which is the root of *Cynanchum auriculatum* Royle ex Wight (Asclepiadaceae) (Peng and Ding 2015). Caudatin has a variety of pharmacological activities including antitumor, immune regulation, antivirus and antioxidation (Zhen et al. 2020). Li et al. (2013) reported that caudatin induced cell apoptosis via Wnt/β-catenin signalling in gastric cancer cells. Besides, caudatin promoted cell cycle arrest and apoptosis in HepG2 cells (Fei et al. 2012). However, the effects of caudatin in lung cancer are still unclear.

Cancer stem cells (CSCs) refer to a small number of cell subsets with self-renewal and unlimited proliferation ability in tumours (Chang 2016). CSCs targeted therapy is of vital importance due to the correlation between CSCs with routine treatment failure, tumour metastasis, recurrence and drug resistance (Yadav and Desai 2019). Sex determining region Y-box 2 (SOX2), octamer binding transcription factor 4 (OCT4) and Nanog are transcription factors participating in the maintenance of pluripotent embryonic stem cell phenotype (Rodda et al. 2005). Highly expressed OCT4, SOX2 and NANOG are closely correlated with advanced disease stage and poor prognosis in cancer (Yang et al. 2018). During the rapid growth of tumour cells, glycolysis is an essential metabolic mode of tumour cell due to insufficient oxygen supply (Ganapathy-Kanniappan and Geschwind 2013). Glycolysis promotes the malignant progress of tumour through providing adenosine triphosphate (ATP) for tumour cell proliferation, invasion and metastasis. Therefore, glycolysis targeted therapy is an important strategy for the treatment of lung cancer (Granchi et al. 2014).

The RAS/MEK/ERK signalling pathway plays major role in cell growth, survival and differentiation (Degirmenci et al. 2020). Inhibition of Warburg effect related signalling pathways in cancer cells has become a promising anticancer strategy. Activation of ERK1 and ERK2 signalling is commonly found in human cancers (Barbosa et al. 2021) and inhibition of ERK signal is a bridge between glycolysis suppression (Papa et al. 2019). The inhibition of MEK weakened the stemness of cancer cells by suppressing sphere and organoid formation capacity and cell migration in pancreatic cancer cells (Walter et al. 2019). Herein, we explored the effects of caudatin on NSCLC cells from the perspective of cell metabolism, including the process of cell proliferation, stemness and glycolysis. We demonstrated that caudatin suppressed the progression of NSCLC cells *in vivo* and *in vitro* by inhibiting cell proliferation, stemness and glycolysis via Raf/MEK/ERK pathway inhibition.

## Materials and methods

### Cell culture

Human lung cancer H1299 and H520 cell lines were purchased from Guangzhou Saiku Biotechnology Co., Ltd. (Guangzhou, China). Human bronchial epithelial cell line BEAS-2B was collected in our laboratory. The cells were kept in in an incubator containing 5% CO_2_ at 37 °C and the RPMI-1640 medium (HyClone, Logan, UT) containing 10% foetal bovine serum (FBS), 100 μg/mL streptomycin and 100 U/mL penicillin.

### Reagents

Caudatin (purity is >98%, cat. no. PHL89596) and LM22B-10 (cat. no. SML2461, purity is ≥98%,), which is the activator of ERK pathway, were purchased from Sigma-Aldrich (St. Louis, MO). Caudatin was dissolved in appropriate amount of dimethyl sulphoxide (DMSO) to make the mother liquor an according to the instructions and the mother liquor was stored at −20 °C away from light. The mother liquor was diluted to the final concentration using cell culture solution before use. To determine the concentration range of caudatin, the H1299 and H520 cells were treated with caudatin at the concentrations of 0, 6.25, 12.5, 25, 50, 100, 200 and 400 μM for 24 h, respectively.

### Cell counting kit-8 (CCK-8) assay

H1299 and H520 cells (2 × 10^3^) were seeded in 96-well plates. Then, 10 μL CCK-8 solution (Beyotime, Shanghai, China) was added to each well (being careful not to generate bubbles in the hole, so as not to affect the accuracy of optical density value) and the plate was incubated for another 1 h. Lastly, the absorbance at 450 nm was detected on a microplate reader (Thermo Fisher Scientific, Boston, MA).

### Cell apoptosis detection

H1299 and H520 cells pre-treated with different concentrations of LQ were harvested and prepared for cell apoptosis detection. The cells were blended with Annexin V-FITC and PI (Sigma, St. Louis, MO) for 15 min at room temperature in dark. Then, the stained cells were analysed through flow cytometry.

### Sphere-forming assay

The sphere-forming assay was conducted with reference to the previous literature (Lu et al. 2020). H1299 and H520 cells were treated with caudatin (purity is >98%) at 0, 25, 50 and 100 μM for 24 h. Then, single cell suspensions were made in stem cell-conditioned medium and 1 × 10^4^ cells were seeded in ultra-low attachment six-well plates (Corning Inc., New York, NY) for 7 d. The number of tumour spheres (sphere diameter over 50 μm) in the six-well plates was counted and photographed. The percentage of spheres formed was calculated using Image J software (v1.8.0, National Institutes of Health, Bethesda, MD).

### Screening of aldehyde dehydrogenase (ALDH) positive cells

ALDH positive cells were screened using the ALDEFLUOR™ kit (cat. no. 01700 Stem Cell Technologies, Shanghai, China) following the manufacturer’s directive rules. ALDEFLUOR reagent and BODIPY-aminoacetaldehyde were added into H1299 and H520 cell suspensions at 37 °C for 40 min. Cells in the control group were treated with ALDH inhibitor ALDEFLUOR DEAB reagent (40 µM) under the same conditions. ALDH^+^ cells were screened on the FACSCanto II system and FACS ARIA software, respectively (BD Biosciences, Bergen, NJ). Dead cells were selected using 1 µg/mL propidium iodide antibody.

### Western blot analysis

Total protein in H1299 and H520 cells was extracted using RIPA buffer (cat. no. 89900, Thermo Fisher Scientific, Boston, MA) and the protein concentrations were determined using BCA protein assay (Pierce, Rockford, IL). Total protein (40 μg) was separated on sodium dodecyl sulphonate-polyacrylamide gel electrophoresis (Boster, Wuhan, China) and was then transferred onto the polyvinylidene difluoride membranes (Millipore, Billerica, MA). After blocking with 1% bovine serum albumin (cat. no. LK-A3828, Multisciences Biotech, Co., Ltd., Shenzhen, China) solution, the membranes were incubated with appropriate primary antibodies at 4 °C overnight and followed by incubating with horseradish peroxidase conjugated secondary antibodies (cat. no. 31430, Thermo Scientific, Waltham, MA) for 1 h at room temperature. β-Actin was used as an internal reference. The band was visualized with enhanced chemiluminescence (Millipore, Billerica, MA) and the film was scanned on the Quantity one software (version 62, Bio-Rad Laboratories, Inc., Hercules, CA).

The primary antibodies (Abcam, Cambridge, UK) used here were: anti-SOX2 (cat. no. ab254193, 1:1000), anti-OCT4 (cat. no. ab181557, 1:1000), anti-Nanog (cat. no. ab109250, 1:1000), anti-hexokinase II (HK2) (cat. no. ab209847, 1:1000), anti-lactate dehydrogenase (LDHA) (cat. no. ab52488, 1:1000), anti-p-Raf (cat. no. ab112053, 1:1000), anti-Raf (cat. no. ab181115, 1:1000), anti-pMEK1/2 (cat. no. ab278723, 1:1000), anti-MEK1/2 (cat. no. ab278564, 1:1000), anti-p-ERK1/2 (cat. no. ab278538, 1:1000), anti-ERK1/2 (cat. no. ab184699, 1:1000) and the anti-β-actin (cat. no. ab8245, 1:2000).

### Evaluation of glycolysis related indexes

Glycolysis intensity was evaluated through detecting glucose uptake, lactic acid production and ATP production. Glucose uptake colorimetric assay kit (cat. no. MAK083, Sigma-Aldrich, St. Louis, MO), lactate assay kit (cat. no. MAK064, Sigma-Aldrich, St. Louis, MO) and the ATP assay kit (cat. no. MAK190, Sigma-Aldrich, St. Louis, MO) were used according to the manufacturer's instructions.

### Evaluation of extracellular acidification rate (ECAR)

The glycolytic flux was further assessed using glycolysis stress test kit (cat. no. 103015-100, Seahorse Bioscience, Lexington, MA) following the manufacturer’s instructions. The cells were seeded in the Seahorse XF cell culture microplates (Seahorse Bioscience, Lexington, MA) at the initial density of 4 × 10^4^ cells/well the day before measurement. The detection solution containing 2 μM oligomycin, 50 mM 2-deoxy-glucose and 10 mM glucose was prepared. The probe card was soaked with the detection solution for one night in an incubator without CO_2_ at 37 °C. The next day, the cells were incubated with the compound in the detection solution following the manufacturer’s instructions. The ECAR value was accessed on the Seahorse XFe24 Extracellular Flux Analyser and computer installed with XFe24 Wave software (Luz et al. 2015).

### Construction of subcutaneous tumour xenografts

Ten pathogen free female nude mice (BALB/C), weighing 13–15 g, 4 weeks old, were purchased from Experimental Animal Center of Jingjiang People’s Hospital (Taizhou, China). The mice were bred in specific pathogen free environment at 25 °C with 50–60% humidity, 12 h illumination time and free access to diet and drinking water. Before experiments, the mice received adaptive feeding for one week. The animal experiments were approved by the animal experimental Ethical Committee of Jingjiang People’s Hospital (no. SYXK (Jiangsu Province) 2018-0013; Taizhou, China) and followed the principles of the Declaration of Helsinki. All nude mice were divided into two groups (*n* = 5). Each nude mouse was injected with 5 × 10^6^ H1299 cells and continued to be fed under normal conditions. The mice in the treatment group received 50 mg/kg/day caudatin through oral gavage and mice in the sham group received equal amount of DMSO. The dose of 50 mg/kg/day used in the animal experiments was selected with reference to the existing drug concentration in the subcutaneous tumour experiment of tumour cells (Tan et al. 2016). The weight of the mice was determined every 5 d and the tumour volume was also determined every 5 d until 30 d following the formula (*a*×*b*^2^)/2 (*a*, *b* are the largest diameter and the perpendicular diameter, respectively). On the 30th day, the nude mice were put to death through rapid cervical dislocation. The tumour was removed completely and tumour weights were assessed and photographed. The tumour was selected for further experiments.

### Caudatin toxicity verification

Ten pathogen free female nude mice (BALB/C), weighing 13–15 g, 4 weeks old, were purchased from Experimental Animal Center of Jingjiang People’s Hospital (Taizhou, China). The mice were raised consistent with that of the tumour model group and the sham group. The animal experiments were approved by the animal experimental Ethical Committee of Jingjiang People’s Hospital (no. SYXK (Jiangsu Province) 2018-0013; Taizhou, China) and followed the principles of the Declaration of Helsinki. Ten mice were divided into two groups randomly (*n* = 5). The mice in the treatment group received 50 mg/kg/day caudatin through oral gavage (keep pace with the dosage in the tumour model group) and mice in the control group received equal amount of DMSO for 30 days. Mice in the control group and caudatin group were fasted for 24 h after the last administration. After weighed and anaesthetized with sodium pentobarbital, the blood was collected through abdominal aorta and the serum was separated at 3000 rpm for 2 min. The serum was kept for further detection.

### Haematoxylin–eosin (HE) staining

The tissues were first fixed in 4% formalin. HE staining kit was used here (cat. no. C0105S, Beyotime Biotechnology, Nanjing, China). After deparaffinization and rehydration, the sections were incubated with haematoxylin solution for 5 min, and then stained in 70% ethanol containing 1% HCl. Then, the sections were stained with eosin solution for 3 min, and then were dehydrated in graded alcohol (70% alcohol for 10 s, 80% alcohol for 10 s, 90% alcohol for 10 s and in absolute ethanol for 10 s, respectively) followed by clearing in xylene for 5 min. At last, the sections were sealed in neutral balsam and representative images were captured using a fluorescence microscope.

### Immunohistochemistry (IHC)

The IHC assay was conducted with reference to the previous literature (Junichi et al. 2010). Paraffin-embedded tumour tissues were cut into 4 µm sections. After dewaxing and rehydrated, the sections received antigen retrieval treatment by submerged in 0.01 M citric acid buffer and microwaved. After the slices were naturally cooled to room temperature, they were rinsed for three times. Then, the sections were blocked in 5% bovine serum albumin solution for 20 min at room temperature. After washing, the sections were incubated with anti-Ki67 (cat. no. ab264429, 1:1000), anti-SOX2 (cat. no. ab97959, 1:1000), anti-OCT4 (cat. no. ab200834, 1:1000), anti-Nanog (cat. no. ab109250, 1:1000), anti-hexokinase II (HK2) (cat. no. ab209847, 1:1000) and anti-lactate dehydrogenase (LDHA) (cat. no. ab52488, 1:1000) (Abcam, Cambridge, MA) antibodies overnight at 4 °C. After washed, the tissue sections were treated with biotinylated anti-rabbit secondary antibody (cat. no. 31466, Thermo Fisher Scientific, Boston, MA) at 37 °C for 1 h. After washing, the sections were incubated with appropriate amount of horseradish enzyme labelled streptomyces ovalbumin working solution at 37 °C for 10–30 min. Then, the sections were stained with 10% Mayer's haematoxylin, dehydrated and sealed with gum. Lastly, the sections were scanned using an Aperio AT2 digital scanner (Leica Biosystems, Wetzlar, Germany).

### Detection about alanine aminotransferase (ALT) and aspartate aminotransferase (AST)

Mice in the sham group and the caudatin group were fasted for 24 h after the last administration. After weighed and anaesthetized with sodium pentobarbital, the blood was collected through abdominal aorta and the serum was separated at 3000 rpm for 2 min. Serum ALT and AST were measured using ALT activity assay kit (Nanjing Jiancheng Bioengineering Research Institute, Nanjing, China, cat. no.: c009-1) and AST activity assay kit (Nanjing Jiancheng Bioengineering Research Institute, Nanjing, China, cat. no.: c010-1).

### Statistical analysis

All the experiments in the study were performed in triplicate. SPSS 13.0 (SPSS Inc., Chicago, IL) and GraphPad Prism 7 (GraphPad Software, La Jolla, CA) were used for statistical analysis. Data were presented as the mean ± SD. The difference between two groups was analysed using an unpaired two-tailed Student’s *t*-test. Comparison between groups was performed by one way analysis of variance followed by the Tukey *post hoc* test. *p* < 0.05 was considered to indicate a statistically significant difference.

## Results

### Caudatin inhibits the proliferation of non-small cell lung cancer cells

**Figure 1.**
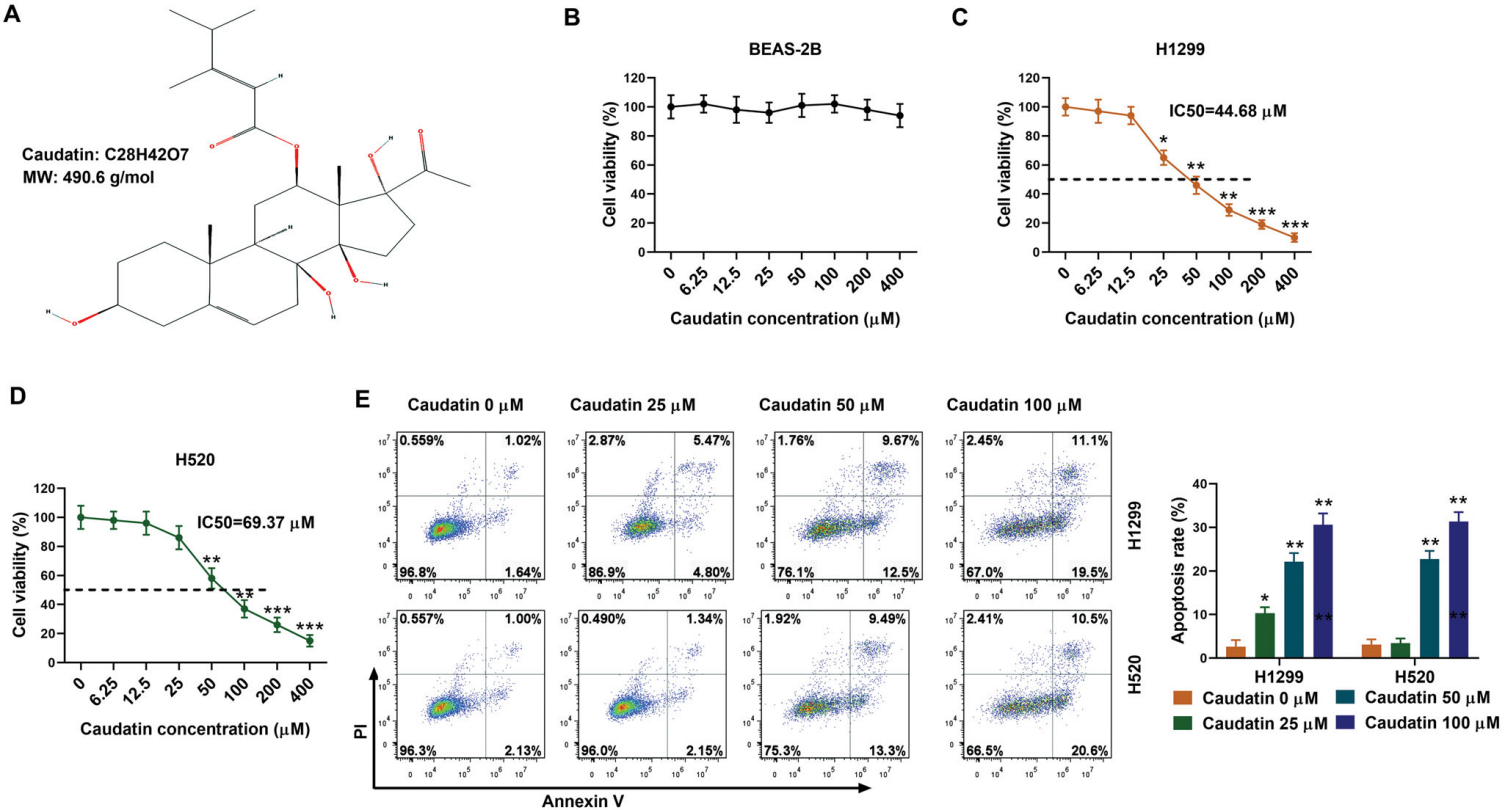
Caudatin inhibits the proliferation of non-small cell lung cancer cells. (A) The molecular structure of caudatin. The H1299 and H520 cells were treated with caudatin at the concentrations of 0, 6.25, 12.5, 25, 50, 100, 200 and 400 μM for 24 h, respectively. (B) BEAS-2B, human normal lung epithelial cells were used here. Effects of caudatin on cell viability in normal lung epithelial cells were verified through CCK8 assay. (C, D) Cell viability of H1299 and H520 cells was detected through CCK8 assay. (E) Cell apoptosis was detected by flow cytometry. The data from triplicate experiments are represented as the mean ± SD. *p < 0.05, **p < 0.01 and ***p < 0.001 vs. the caudatin 0 μM group.

Rapid proliferation is a characteristic of malignant tumour cells. The molecular structure of caudatin was first displayed as in Figure 1(A). The H1299 and H520 cells were treated with caudatin at the concentrations of 0, 6.25, 12.5, 25, 50, 100, 200 and 400 μM for 24 h. BEAS-2B, human normal lung epithelial cells were used here. The results showed that caudatin had no obvious effects on cell viability in normal lung epithelial cells (Figure 1(B)), indicating that caudatin had no obvious cytotoxicity. The cell viability of H1299 and H520 cells was both destroyed by caudatin, with the 50% inhibitory concentration (IC_50_) of 44.68 and 69.37 μM, respectively (Figure 1(C,D)). Cell apoptosis was also notably enhanced with increased concentration of caudatin (Figure 1(E)). Thus, caudatin efficiently inhibits the proliferation of NSCLC cells.

### Caudatin inhibits the stemness of non-small cell lung cancer cells

**Figure 2.**
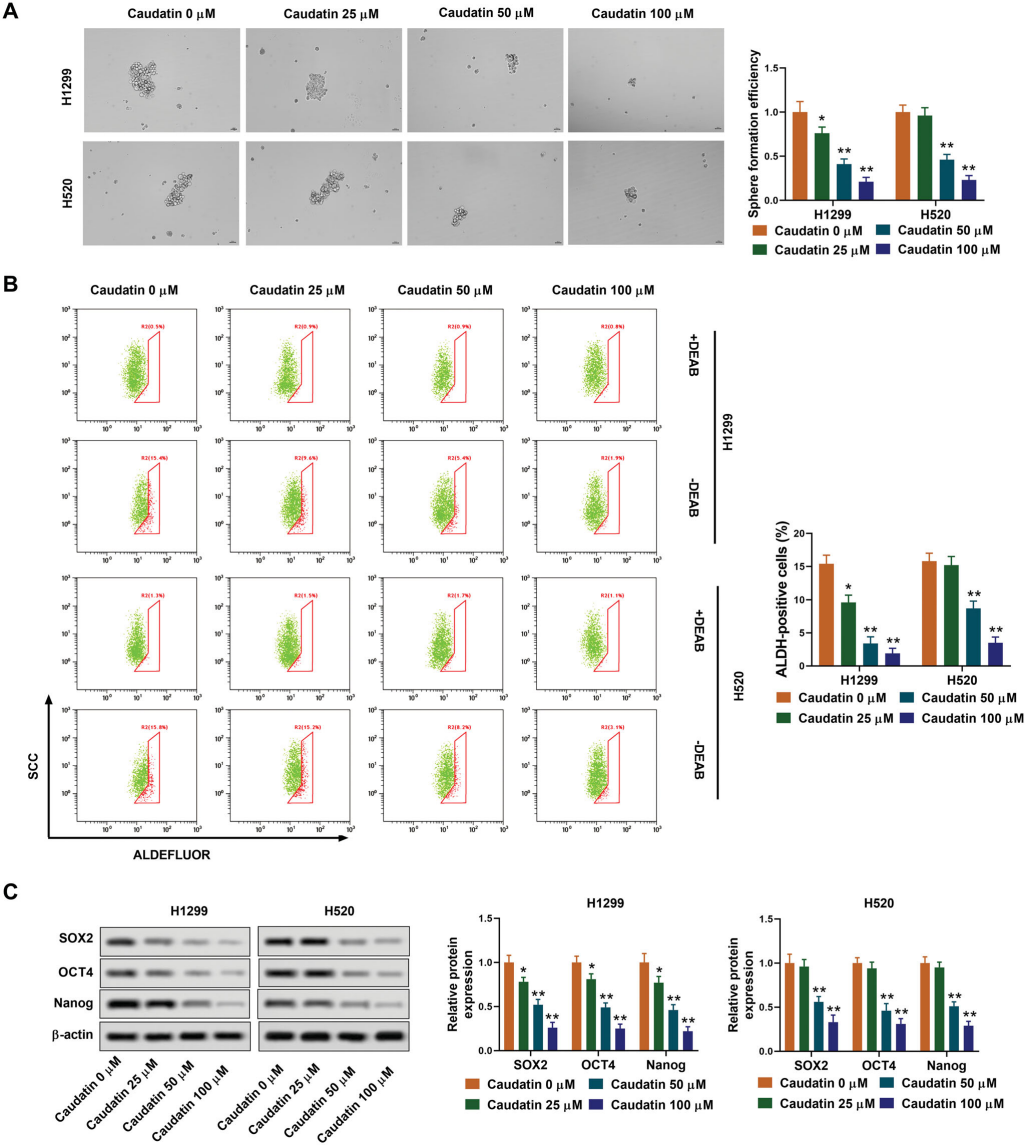
Caudatin inhibits the stemness of non-small cell lung cancer cells. Caudatin at the concentrations of 0, 25, 50 and 100 μM was selected for the following experiments. (A) Sphere formation efficiency of H1299 and H520 cells was valued by sphere-forming assay. (B) ALDH positive cells were selected by flow cytometry. (C) The expression of tumour stem cell related factors SOX2, OCT4 and Nanog was detected through western blotting. The data from triplicate experiments are represented as the mean ± SD. *p < 0.05 and **p < 0.01 vs. the caudatin 0 μM group.

Caudatin at the concentrations of 0, 25, 50 and 100 μM was selected for the following experiments. As we could see, sphere formation efficiency of H1299 and H520 cells was strongly weakened by caudatin (Figure 2(A)). Besides, the ratio of ALDH positive cells was also sharply decreased with increasing concentration of caudatin, indicating that the number of stem cell in H1299 and H520 cells was decreased by caudatin treatment (Figure 2(B)). At the same time, the expression of tumour stem cell related factors (SOX2, OCT4 and Nanog) was suppressed in the existence of caudatin (Figure 2(C)). Thus, stemness of NSCLC cells was also weakened by caudatin.

### Caudatin inhibits glycolysis in non-small cell lung cancer cells

**Figure 3.**
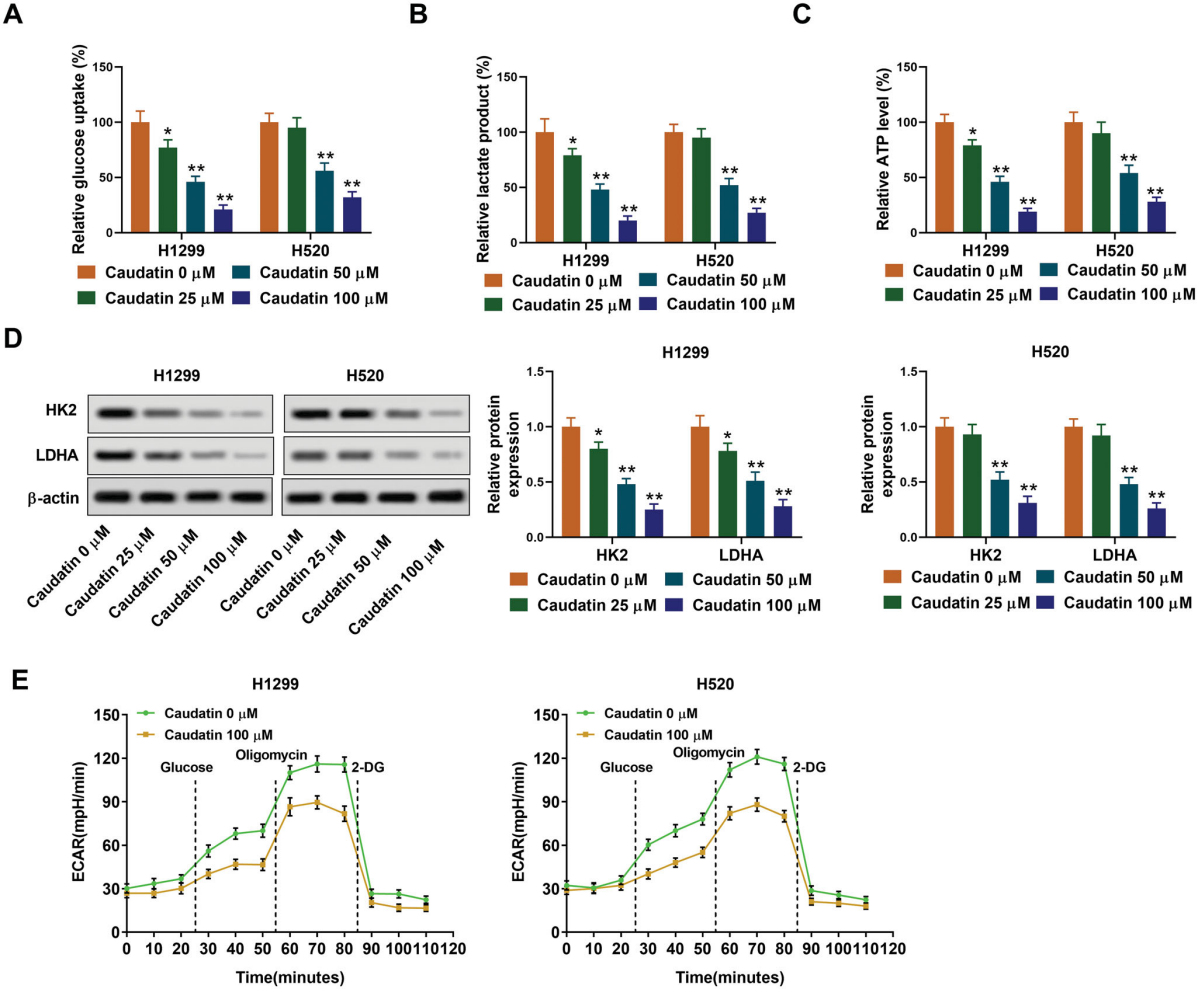
Caudatin inhibits glycolysis in non-small cell lung cancer cells. (A–C) Glucose uptake, lactic acid production and ATP level in H1299 and H520 cells were detected using corresponding kits. (D) The expression of glycolysis related proteins HK2 and LDHA was detected through western blotting. (E) ECAR was further assessed using glycolysis stress test kit on the Seahorse XFe24 Extracellular Flux Analyser and computer installed with XFe24 Wave software. The data from triplicate experiments are represented as the mean ± SD. *p < 0.05 and **p < 0.01 vs. the caudatin 0 μM group.

Glycolysis pathway is the main energy supply mode of tumour cells, is also an important research target to curb the proliferation of tumour cells. As shown in Figure 3(A–C), glucose uptake, lactic acid production and ATP level in H1299 and H520 cells were sharply decreased by caudatin treatment. At the same time, the expression of glycolysis related proteins HK2 and LDHA was also decreased with increasing concentration of caudatin (Figure 3(D)). In addition, ECAR, the extracellular acid production ability of cells which indirectly reflected the glycolysis rate, was detected. The results showed that glycolysis rate was sharply reduced in caudatin 100 μM group of H1299 and H520 cells (Figure 3(E)). These above results demonstrated that cell glycolysis was effectively suppressed by caudatin.

### Caudatin inhibits the malignant phenotype of non-small cell lung cancer cells by Raf/MEK/ERK pathway inhibition

**Figure 4.**
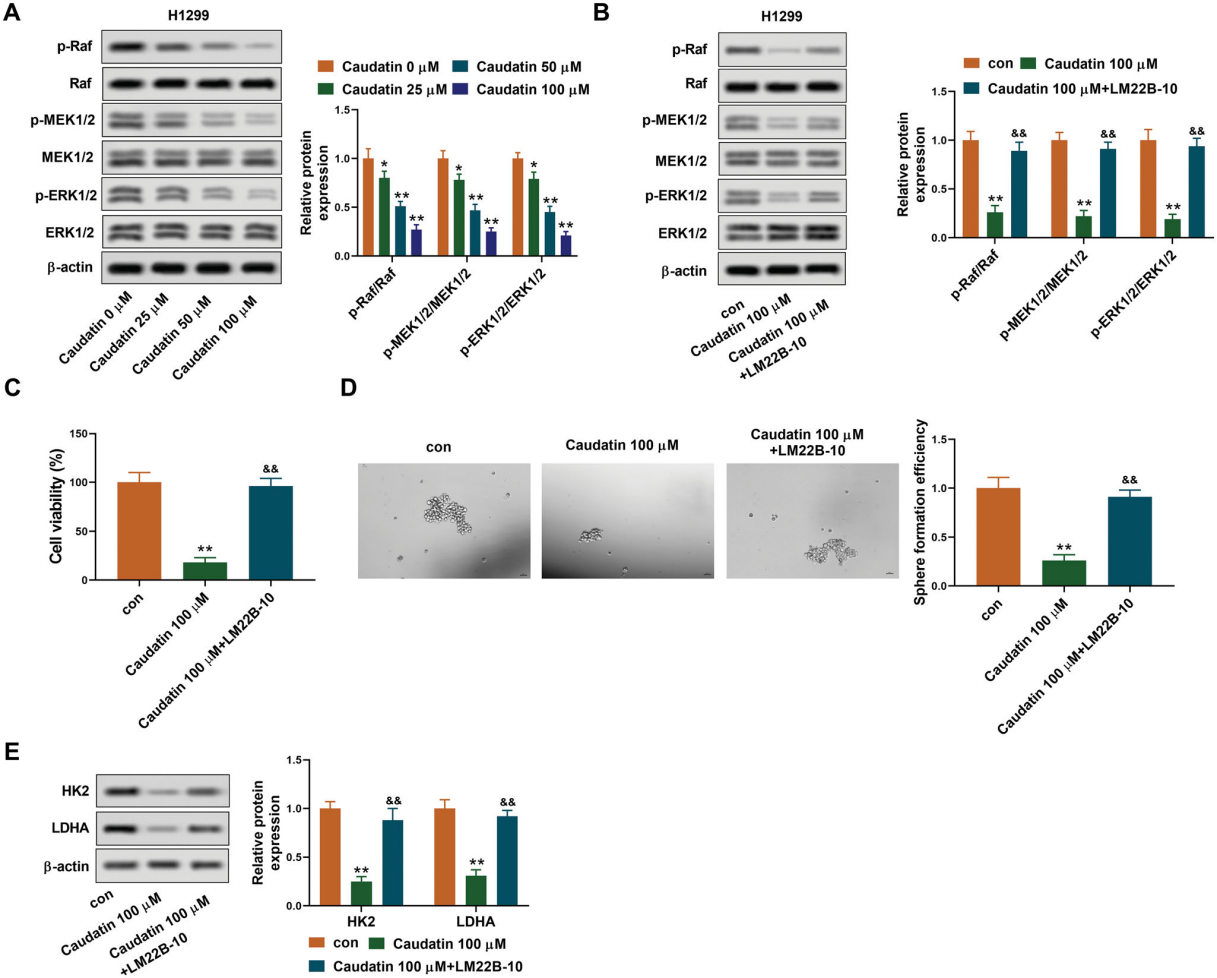
Caudatin inhibits the malignant phenotype of non-small cell lung cancer cells by Raf/MEK/ERK pathway inhibition. The associated signal pathway was also explored. (A) The protein levels of Raf/MEK/ERK pathway related p-Raf/Raf, pMEK1/2/MEK1/2 and p-ERK1/2/ERK1/2 were detected through western blotting. The ERK pathway activator LM22B-10 (5 mM) was used to activate the Raf/MEK/ERK pathway in the following experiments. The H1299 cells were grouped as control group, caudatin 100 μM group and caudatin 100 μM group + LM22B-10 group. (B) The protein levels of p-Raf/Raf, pMEK1/2/MEK1/2 and p-ERK1/2/ERK1/2 were detected through western blotting. (C) Cell viability of H1299 and H520 cells was detected through CCK8 assay. (D) Sphere formation efficiency of H1299 cells was valued by sphere-forming assay. (E) The expression of glycolysis related proteins HK2 and LDHA was detected through western blotting. The data from triplicate experiments are represented as the mean ± SD. *p < 0.05 and **p < 0.01 vs. the control group. ^&&^*p* < 0.01 vs. the caudatin 100 μM group.

The associated signal pathway was also explored. The protein levels of p-Raf, pMEK1/2 and p-ERK1/2 decreased gradually with the increase of caudatin concentration, and the same is the ratio of p-Raf/Raf, pMEK1/2/MEK1/2 and p-ERK1/2/ERK1/2 (Figure 4(A)). Thus, the Raf/MEK/ERK pathway was inactivated by caudatin. To further verify the effects of Raf/MEK/ERK pathway, the ERK pathway activator LM22B-10 (5 mM) was used to activate the Raf/MEK/ERK pathway. As shown in Figure 4(B), the protein levels of p-Raf, pMEK1/2 and p-ERK1/2 were elevated in the existence of LM22B-10 compared with caudatin 100 μM group, indicating that the Raf/MEK/ERK pathway was successfully activated by LM22B-10. We found that the inhibitory effect of caudatin on cell viability and sphere formation efficiency was largely weakened by the combined action of LM22B-10 (Figure 4(C,D)). The suppressed expression of HK2 and LDHA by caudatin was rescued by the adding of LM22B-10, indicating that the activation of Raf/MEK/ERK pathway neutralized the inhibiting effect of caudatin on malignant phenotype (Figure 4(E)). The above results verified that caudatin suppressed cell proliferation, stemness and glycolysis by Raf/MEK/ERK pathway inhibition.

### Caudatin inhibits the proliferation of non-small cell lung cancer cells *in vivo*

**Figure 5.**
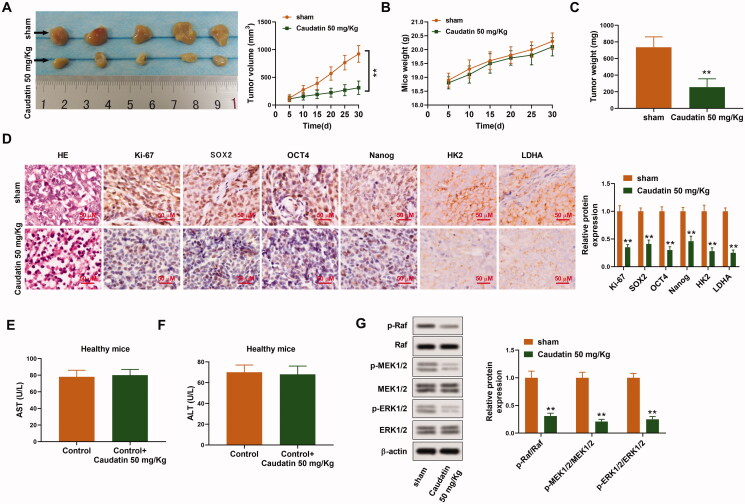
Caudatin inhibits the proliferation of non-small cell lung cancer cells *in vivo*. The subcutaneous tumour model of human lung cancer was established by intraperitoneal injection of H1299 cells (5 × 10^6^). The mice in the treatment group were treated by caudatin at the dose of 50 mg/kg. (A) Tumour picture presentation and the analysis of the tumour volume. (B, C) Comparison of mice weight and tumour weight between the treatment group and the sham group. (D) The HE staining and the expressions of Ki-67, SOX2, OCT4, Nanog, HK2 and LDHA in tumour tissues were valued using IHC assay (scale bar, 50 μM). (E, F) Ten healthy mice in the experimental group and the control group were treated with 50 mg/kg caudatin (*n* = 5) or DMSO (*n* = 5), respectively. Serum ALT and AST in healthy mice were measured using ALT activity assay kit and AST activity assay kit, respectively. (G) The protein levels of p-Raf/Raf, pMEK1/2/MEK1/2 and p-ERK1/2/ERK1/2 were detected through western blotting. The data from triplicate experiments are represented as the mean ± SD. **p < 0.01 vs. sham group.

We further conducted the following *in vivo* experiments to provide basis for the clinical application of caudatin. The subcutaneous tumour model of human lung cancer was established by intraperitoneal injection of H1299 cells (5 × 10^6^). The mice in the treatment group were treated by caudatin at the dose of 50 mg/kg. Representative pictures of the tumour in Figure 5(A) showed that tumour volume of caudatin 50 mg/kg group was much smaller than that in the sham group. There was no significant difference between the mouse weight in the two groups (Figure 5(B)). In addition, much smaller tumour weight was found in caudatin group than that in the sham group (Figure 5(C)). HE staining showed that the cells in the sham group had different sizes and shapes and the nucleocytoplasmic ratio increased. Besides, the chromatin was granular and unevenly distributed. The cells in the caudatin 50 mg/kg group showed more morphological changes related to apoptosis, such as chromatin aggregation and nuclear pyknosis (Figure 5(D)). In addition, the expression of Ki-67, SOX2, OCT4, Nanog, HK2 and LDHA in the caudatin 50 mg/kg group was much lower than that in the sham group which was detected through IHC (Figure 5(D)). In order to eliminate the interference of the toxic effect of caudatin on the experimental results, the content of serum AST and ALT in healthy mice was detected. Total of 10 healthy mice were used here for the safety examination. The mice in the caudatin group receive caudatin treatment at the dose of 50 mg/kg/d, and the mice in the control group received equal amount of DMSO for 30 days. No obvious difference existed between the level of AST and ALT in the two groups, indicating that toxic effect of caudatin is negligible in our current experiments (Figure 5(E,F)). Furthermore, the Raf/MEK/ERK pathway was also inhibited *in vivo* by caudatin treatment, presenting as decreased ratio of p-Raf/Raf, pMEK1/2/MEK1/2 and p-ERK1/2/ERK1/2 (Figure 5(G)). Thus, caudatin inhibits the proliferation of NSCLC cells *in vivo*.

## Discussion

According to the latest data released by National Central Cancer Registry of China in 2015, lung cancer has the highest incidence rate and mortality in China (Chen et al. 2016). Although progress has been achieved in the treatment of lung cancer, the overall 5-year survival rate of lung cancer patients is only 4–17% (Siegel et al. 2016). According to a large number of tumour treatment cases, TCM combined with traditional radiotherapy and chemotherapy can significantly alleviate the adverse reactions of drugs and improve the therapeutic effect (Qi et al. 2015). Therefore, it is of great significance to study the molecular mechanism of active components of TCM in lung cancer.

Caudatin is a C-21 steroidal glycoside with various pharmacological activities isolated from the root of *Cynanchum auriculatum* (Zhu et al. 2016). In recent years, more and more studies have shown that caudatin and its derivatives have significant antitumor effects. For example, Wang et al. (2013) reported that caudatin resulted in G1 phase cell cycle arrest and obvious apoptosis in gastric cancer SGC-7901 cells. Luo et al. (2013) reported that caudatin blocked cell growth and metastasis in human hepatoma cells via modulating the Wnt/β-catenin pathway. However, the effects of caudatin on lung cancer and the associated mechanism are still not completely clear.

Cancer stem cells have the abilities of unlimited proliferation, self-renewal and multidirectional differentiation, which easily induce tumour residue and recurrence (Yanamoto et al. 2014). Besides, CSCs weaken the efficacy of radiotherapy through DNA damage repair and cytoprotective autophagy, resulting in poor prognosis (Lin et al. 2015; Vitale et al. 2017). Thus, CSCs related biological markers have been taken as therapeutic targets in cancer treatment (Sun JH et al. 2016; Eun et al. 2017). ALDH1A1, SOX2, NANOG and OCT-4 are all acknowledged biomarkers of lung cancer (Lu et al. 2020; Masciale et al. 2020). Overexpressed OCT4 always presages tumour occurrence, metastasis, recurrence and drug resistance (Jen et al. 2017). Aldehyde dehydrogenase participates in the self-protection, differentiation and expansion of CSCs, and affects the prognosis of malignant tumours (Tomita et al. 2016). Liu X et al. (2016) reported that ALDH1A1 inhibition suppressed the recurrence of NSCLC driven by ALDH-positive CSCs. Yang et al. (2021) reported that XMD-17-51 blocked the progression of lung cancer by reducing the expression of SOX2, NANOG and OCT4. In our study, caudatin treatment markedly suppressed cell viability and induced cell apoptosis. Caudatin treatment effectively weakened the sphere formation efficiency of H1299 and H520 cells and decreased the ratio of ALDH positive cells, indicating that cell stemness was sharply weakened. At the protein level, the expression of SOX2, OCT4 and Nanog was all down-regulated by caudatin. Thus, the antitumor effects of caudatin on NSCLC were related to stemness inhibition.

Tumour cells have the characteristics of rapid proliferation, during which a large amount of energy is consumed rapidly. In the 1920s, Otto Warburg found that glycolysis was an important energy source for the rapid proliferation tumour cells, which is also called as Warburg’s effect (Liberti and Locasale 2016). Warburg effect is manifested as notably elevated glucose uptake, lactate production and ATP production in tumour cells. Lactic acid produced in the process of glycolysis is conducive to the growth of tumour cells (Sun S et al. 2017). Elevated lactic acid production promotes the angiogenesis, migration, prognosis and recurrence of tumour (Colegio 2016). Thus, targeting lactate metabolism is a promising and effective approach for cancer treatment (Doherty and Cleveland 2013). In our study, glucose uptake, lactic acid production and ATP level in H1299 and H520 cells were sharply decreased by caudatin treatment. GLUT1, HK2, PKM2 and LDHA are all glycolysis related genes (Liu R et al. 2019). Wu et al. (2020) reported that inhibition of key glycolytic genes blocked the progression of gastric cancer. In the present study, the expression of HK2 and LDHA was also decreased with increasing concentration of caudatin, indicating that caudatin effectively suppressed the proliferation of LSCC cells by inhibiting glycolysis. Extracellular acidification rate is an important indicator of the metabolism in cells (Villani et al. 2016). In our study, caudatin substantially decreased the level of ECAR, which indirectly reflected the glycolysis rate. In general, caudatin effectively weakened glycolysis in lung cancer cells.

The Raf/MEK/ERK pathway is an important signal transduction pathway which is closely related to tumorigenesis, cell proliferation, differentiation and apoptosis (Asati et al. 2016). Hyper-activated Raf/MEK/ERK pathway is commonly found in tumours and the use of compounds targeting components has promising clinical activity in cancer treatment (Samatar and Poulikakos 2014). Abnormal activation of Raf/MEK/ERK pathway is an indication for cancer progression. In our study, the Raf/MEK/ERK pathway was inactivated by caudatin, presenting as decreased ratio of p-Raf/Raf, pMEK1/2/MEK1/2 and p-ERK1/2/ERK1/2. In addition, the inhibitory effects of caudatin on cell proliferation, stemness and glycolysis were neutralized by LM22B-10, which acts as the activator of Raf/MEK/ERK pathway. The above results indicated that activated Raf/MEK/ERK pathway promoted cell proliferation, stemness and glycolysis. As previously reported, homeodomain-interacting protein kinase 2 blocked the proliferation and aerobic glycolysis in pancreatic cancer cells via ERK/cMyc signal inhibition (Qin et al. 2019). Thus, the present study verified that caudatin suppressed cell proliferation, stemness and glycolysis by Raf/MEK/ERK pathway inhibition. In future studies, we will further assess the effects of caudatin on cell mobility in NSCLC cells. Besides, most cancer cells still have mitochondrial function, so glycolysis targeted therapy is not the only way. We will further explore alternative anti-metabolism treatments, such as targeting mitochondrial metabolism, inhibiting pentose phosphate pathway, inhibiting fatty acid synthesis and so on. A deeper understanding of metabolic changes and available targets about stemness in tumour cells will help to develop and select effective anticancer drugs.

Apart from this, the *in vivo* experiments were conducted to verify the effects of caudatin on NSCLC. Tan et al. (2016) reported that caudatin significantly decreased tumour size, overall tumour weight and mean tumour volume. Similarly, tumour volume, mice weight and tumour weight of subcutaneous NSCLC tumour model were effectively suppressed by caudatin. In addition, HE staining showed that the caudatin treatment induced more obvious cell apoptosis. The expression of Ki-67, SOX2, OCT4, Nanog, HK2 and LDHA in the caudatin 50 mg/kg group was much lower than that in the sham group. Similar to the *in vitro* experiments, Raf/MEK/ERK pathway was inactivated in tumour tissues which were examined in the *in vivo* experiments. The safety of caudatin was verified in healthy mice. Serum AST and ALT level had no obvious difference between the caudatin treatment group and the control group in healthy mice, which made it clear that the dosage of caudatin is non-toxic in our experiments. Caudatin inhibits the proliferation of NSCLC cells *in vivo*.

## Conclusions

In this study, caudatin suppressed the proliferation of NSCLC cells, stemness and glycolysis *in vivo* and *in vitro*. The antitumor effects of caudatin were associated with the inactivation of the Raf/MEK/ERK pathway. Our present study proved the antitumor effects of caudatin on NSCLC from the aspects of stemness and glycolysis for the first time, providing a clue for the application of TCM in the treatment of lung cancer.

## Data Availability

The datasets used or analysed during the current study are available from the corresponding author on reasonable request.
